# Current Strategies in Cardiovascular Biomaterial Functionalization

**DOI:** 10.3390/ma3010638

**Published:** 2010-01-22

**Authors:** Simon Schopka, Thomas Schmid, Christof Schmid, Karla Lehle

**Affiliations:** 1 Department of Cardiothoracic Surgery, University Medical Center Regensburg, D-93042 Regensburg, Germany; E-Mails: Christof.Schmid@klinik.uni-regensburg.de (C.S.); Karla.Lehle@klinik.uni-regensburg.de (K.L.); 2 German Aerospace Center, Institute of Robotics and Mechatronics Robotic and Mechatronic Robotic Systems, D-82234 Oberpfaffenhofen-Wessling, Germany; E-Mail: Thomas.Schmid@dlr.de

**Keywords:** tissue engineering, cardiovascular, biomaterials, biofunctionalization

## Abstract

Prevention of the coagulation cascade and platelet activation is the foremost demand for biomaterials in contact with blood. In this review we describe the underlying mechanisms of these processes and offer the current state of antithrombotic strategies. We give an overview of methods to prevent protein and platelet adhesion, as well as techniques to immobilize biochemically active molecules on biomaterial surfaces. Finally, recent strategies in biofunctionalization by endothelial cell seeding as well as their possible clinical applications are discussed.

## 1. Introduction

Cardiovascular disease (CVD) accounted for 16.7 million, or 29.2%, of total global deaths according to the 2003 World Health Report. By 2010, CVD will be the leading cause of death worldwide [http://www.who.int/entity/whr/2004/annex/topic/en/annex_2_en.pdf]. Therefore, the need for improved cardiovascular healthcare is large and growing. This includes a permanently growing demand for polymeric biomaterials as effective implants and graft material. These applications range from indwelling catheters, circuit lines for hemodialysis, extracorporeal circulation to vascular and heart valve prosthesis and ventricular assist devices. The main problem for successful clinical application is formation of clots and induction of thrombotic events after contact of blood with foreign biomaterial surfaces. The prevention of thrombotic deposition and occlusion, triggered by the activation of the coagulation cascade and platelets, is mandatory for an adequate bio- and hemocompatibility and for the functional integrity of these applications. 

Three main strategies to improve hemocompatibility of biomaterials are currently discussed in the literature: (1) surface modification to inhibit blood-material interaction [1], (2) bioactive coatings to achieve active antithrombotic functions [2,3], and (3) endothelialization of blood-contacting surfaces [4].

The present review emphasizes the principle mechanisms of blood contact and platelet activation. Promising antithrombotic strategies are discussed. Special attention was laid on the *in situ* endothelialization of artificial surfaces by immobilizing endothelial cells or endothelial progenitor cells after biomaterial implantation. 

## 2. Blood Contact Activation

Thrombus formation on biomaterial surfaces is a complex network of processes including platelet-mediated reactions and the coagulation of blood plasma itself. Thrombogeneity of the biomaterial depends on the surface chemistry of the biomaterial and on characteristics of blood flow in which the biomaterial is immersed [1]. Vogler and Siedlecke [1] used a simplified model to study the contact activation of blood-plasma coagulation. They postulated that “the activation of the plasma-coagulation cascade is apparently catalyzed by contact of certain blood factors with surfaces. This contact does not necessarily require adsorption of these factors.” 

**Figure 1:**
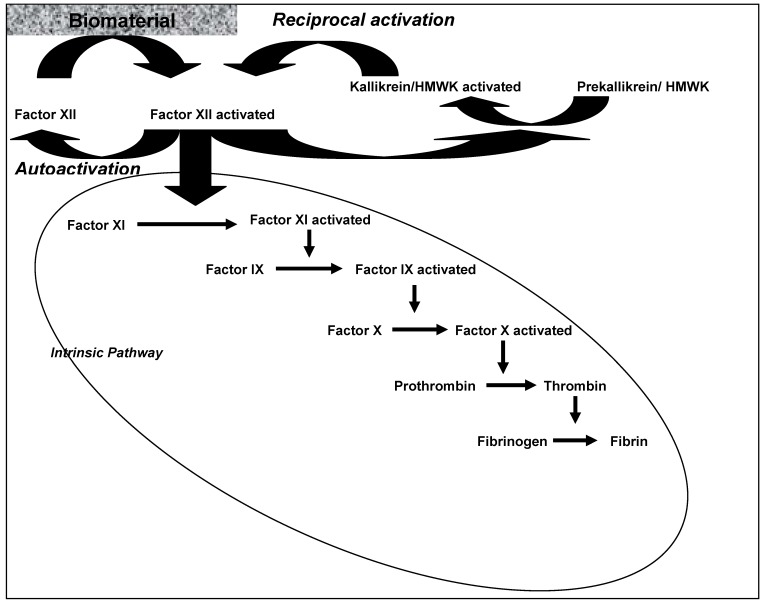
Activation of the coagulation system is initiated by biomaterial-protein interaction. Activation of factor XII is the initial step. Reciprocal activation and autoactivation lead to amplification of activated factor XII, which in turn initiates the intrinsic coagulation pathway *via* activation of factor XI, leading ultimately to the production of fibrin (details see in the text).

As shown in Figure 1, the plasma coagulation consisted of a series of interconnected self-amplifying, zymogen-enzyme conversions. The plasma-coagulation cascade consists of two separate initial pathways (intrinsic and extrinsic) that can be separately potentiated but converge on a common pathway leading to the generation of thrombin (FII). Under normal physiological conditions the extrinsic pathway is responsible for hemostatic control and for the response to vascular injury. The cascade of the extrinsic route is not part of this review. The intrinsic pathway is a complex process that is activated by molecular interactions at the blood-material surface and implements a series of limited proteolytic conversions of zymogens to active enzymes [5,6] (Figure 1). This process might be responsible for poor bio-/hemocompatibility of cardiovascular biomaterials [7,8]. The initial step of the intrinsic pathway is surface-contact activation of the blood zymogen FXII (Hageman factor) into an active enzyme form FXIIa (“autoactivation”). Binding of FXII to negatively-charged surfaces *via* “specific interactions” leads to the assembly of an activation complex involving FXIIa and the allosteric proteins prekallikrein (PK, Fletcher factor), high-molecular weight kininogen (HK, Williams-Fitzgerald factor, HMWK), and FXI (thromboplastin antecedent). Reciprocal-activation of FXII and prekallkrein and cascade propagation by FXIIa-mediated FXI hydrolysis were also described as biochemical reactions to produce FXIIa [1] However, the exact nature of the FXII adsorption/binding/contact step is unclear. Furthermore, the participation of protein adsorption in the contact activation remains a contentious subject. It was discussed that the protein composition of the fluid phase is an essential component of blood plasma coagulation (protein-adsorption-competition effect) (cf. ref. [1]). 

## 3. Platelet Activation

**Figure 2:**
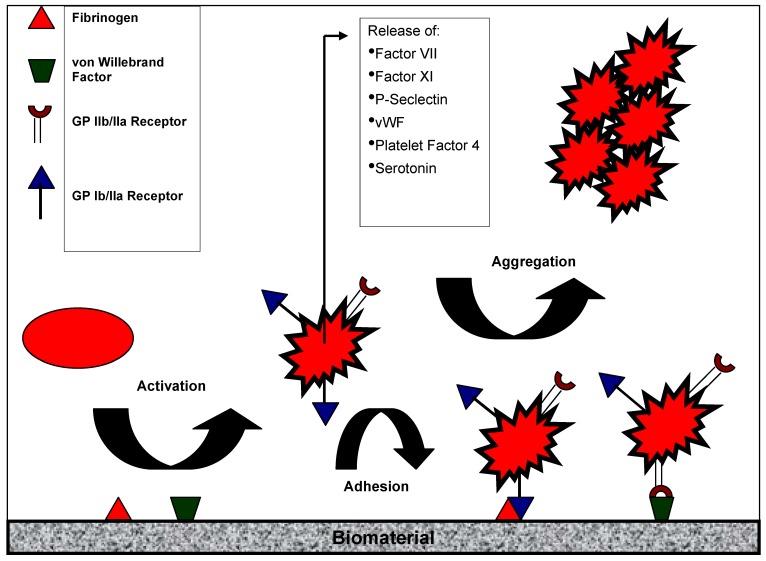
Activation of platelets by artificial surfaces. Contact of platelets with artififcial surfaces leads to platelet activation in terms of ligand expression (GP IIb/IIIa). Activated platelets either adhere to the sufaces (*via* proteins like fibrinogen) or aggregate (details see text).

Thrombogenesis is the basic problem when blood comes in contact with biomedical devices (e.g., cardiopulmonary bypass, hemodialysis, vascular grafts, catheters). Blood-biomaterial contact induces platelet activation (platelet release, P-selectin expression, aggregation) and adhesion [9,10] ending in the formation of a thrombus. Platelet biology and the role of platelets in biomaterial-associated thrombosis were reviewed by Gorbet and Sefton [11]. The following chapter summarizes the main mechanisms of platelet activation after blood-biomaterial contact (Figure 2).

Platelets become activated after contact with any thrombogenic surfaces such as injured endothelium, subendothelium and also artificial surfaces. The first event when a medical device comes in contact with blood is the adsorption of proteins including vitronectin, fibronectin, von Willebrand Factor (vWF) and fibrinogen (Fg) [11] – a process that takes only milliseconds. These proteins interact with specific receptors on the platelet plasma membrane. Among the different platelet adhesion receptors, glycoprotein GPIb and GPIIb/IIIa have the highest density on platelets. GPIb binds to adsorbed or immobilized vWF on the surface, while the active form of GPIIb/IIIa crosslinkes with Fg. Upon platelet activation, a conformational change occurs leading to the upregulation of high-affinity binding sites for adhesion proteins. Binding of Fg or other glycoproteins containing Arg-Gly-Asp (RGD) sequences to activated GPIIb/IIIa leads to platelet aggregation. Activated platelets also release intracellular granules containing coagulation factors VII and XI, the adhesion molecules P-selectin and vWF, serotonin and platelet factor 4, all of which impact further the activation of other platelets, coagulation and inflammation [12]. 

The idea to create non-thrombogenic surfaces by prevention of protein and platelet adhesion has not, unfortunately, proved that simple. It was shown that blood-biomaterial contact could activate platelets leading to removal from circulation rather than adherence on the surface. Furthermore microemboli were formed rather than occlusive thrombi. It was speculated that after contact with adsorbed plasma coagulation proteins, platelets will either adhere or rebound [13], depending on their state of activation and the ligands present at the interface [14]. The usage of hydrogels which were characterized by reduced protein (albumin, Fg, IgG) adsorption capacity [15] prevents platelet adhesion but does not preclude platelet activation as shown by the generation of platelet microparticles [16]. Furthermore, animal studies have shown that, despite the absence of platelet adhesion, blood contact with various hydrogel surfaces [17,18,19] appears to activate platelets, resulting in their removal from the circulation. In this context, the effectiveness of Fg as a main platelet agonist *in vivo* is not clear [20,21]. One more question remained: which factor activates the platelet to allow initial adherence?

The mechanism of material-induced platelet activation is often presumed to be *via* the generation of thrombin due to activation of the intrinsic coagulation cascade (as discussed above) or the release of adenosine diphosphate (ADP) from damaged red blood cells or platelets. Even in the presence of heparin, small levels of thrombin are generated and may activate platelets. However, the inability of thrombin and kallikrein inhibitors to reduce platelet activation suggests that platelet activation is at least in part mediated by other agonists [22]. Gorbet and Sefton [11] discussed a correlation between complement activation and thrombocytopenia which has been noted during dialysis [23,24].

## 4. Strategies to Prevent Protein and Platelet Adhesion

As shown above, protein adsorption on biomaterial surfaces increased both blood contact activation and platelet activation causing thrombotic and thromboembolic complications. Therefore, cardiovascular tissue engineering focused on surface modifications to prevent blood protein adsorption. A summary of recent progress in generating synthetic thromboresistant surfaces that inhibit protein and cell adsorption, thrombin and fibrin formation, and platelet activation and aggregation was composed by Jordan and Chaikoff [25]. In our present review we focused on the biofunctionalization of biomaterials *via* endothelialisation. The next chapter includes only a short overview of current approaches and refer readers to previous extensive reports (Table 1). 

The rationale for the inhibition of protein and cell adsorption is the generation of hydrophobic, chemically inert surfaces or surface coatings, which imitate the so called “lotus effect”. Promising materials are presented in Table 1. The most known polymer is polyethylene oxide (also PEG, polyethylene glycol) [26,27] whose hydrophilic ether oxygen in its structural repeat unit seems to be responsible for its reduced protein binding capacity. However, *in vivo* application and clinical studies so far presented less convincing results. Pyrolytic, graphitic, and carbon coatings are also commonly used materials for clinical applications (vascular grafts, heart valves, vascular stents). For example, carbon-coated vascular vessel grafts implanted in animals improved patency rates [28], and reduced platelet adhesion [18] compared to uncoated materials. 

**Table 1:** Strategies to improve biomaterial hemocompatibility by inhibiting protein and cell adsorbtion, thrombin and fibrin formation and platelet activation.

Strategy	Material	Laboratory success	Clinical success	References
Inhibition of protein and cell adsorbtion	Polyethylene oxide Pyrolytic carbon coating Phosphorycholine surfaces Elastin inspired polymer surfaces	+ + + +	- - - -	[26,27] [28,29,30,31,32,33,34,35] [36,37,38,39,40] [41,42]
Inhibition of thrombin and fibrin formation	Heparin Thrombomodulin	+ +	+ -	[2,3,44,45,46,47,48,49,50] [51,52,53]
Inhibition of platelet activation	Antiplatelet drugs	+	+	[54]

Furthermore, carbon-coating prevented early thrombotic occlusion after coronary stent implantation. However, none of the implanted carbostents improved the long-term outcome in animal studies [29] and in clinical trials [30,31,32,33,34]. Another strategy is the development of stable “membrane-mimetic” films using the protein-repelling properties of the phospholipid monolayer (phosphorylcholine) the main component of biological membranes [36,37,38,39,40]. Different methods such as implementation of protein anchors, heat stabilization, and in situ polymerization of synthetically modified polymerizable phospholipids were used to improve stability of these coatings. Recently, phosphorylcholin-coated polymers were used as vascular vessel grafts in animals with excellent blood compatibility [40]. However, no randomised prospective trial approved the unique benefit of phosphatidylcholine coatings. Finally, the anti-thrombotic properties of elastin, a constituent structural protein of the vessel wall [41], were used to impregnate polymeric vascular vessel grafts with a recombinant elastin and implant this construct in a baboon extracorporeal femoral arteriovenous shunt model [42]. Short-term blood-contact resulted in minimal fibrin and platelet deposition. Furthermore, under *in vitro* conditions, elastin-coatings also reduced fibrinogen and immunoglobulin adsorption as well as the release of proinflammatory cytokines by monocytes [43]. However, clinical data failed.

Inhibition of thrombin and fibrin formation is mediated *via* attachment of biochemically active molecules such as heparin [2,3,44,45,46,47,48,49,50] or thrombomodulin [51,52,53]. The best-known technique is heparin-coating, which finds broad clinical acceptance in cardiopulmonary bypass circuits [2,3]. Differential immobilization strategies were used in experimental and clinical studies to demonstrate an improved thromboresistance and a better platelet preservation using heparin-coated equipments (as reviewed by [2]). In a clinical trial with 300 patients covalent and ionic-bonded heparin-coated cardiopulmonary perfusion circuits reduced the rate of thrombin formation, protected platelets, and reduced postoperative bleeding or transfusion requirements [2]. However, despite a reduction in the rate of thrombin formation, protection of platelets, and reduction of postoperative bleeding or transfusion requirements, there was no benefit on the long-term outcome of the patients [2]. Immobilization of thrombomodulin onto polymeric surfaces should limit the local generation of thrombin. First experiments showed promising short-term results [52]. Under *in vitro* conditions, human thrombomodulin-immobilized nitinol surfaces showed reduced platelet adhesion properties which could improve the blood compatibility of nitinol [53]. However, immobilization of thrombomodulin could reduce its bioactivity. An additional strategy is the production of membrane-mimetic surface assemblies containing thrombomodulin that displayed prolonged stability and activity in high shear environment [25]. The clinical impact of effective thromboresistant thrombomodulin-coating has to be examined in a prospective clinical study. The immobilization of platelet inhibitors might be a prospective approach in cardiovascular tissue engineering. Kidane *et al.* [54] reviewed the current state of the art of antiplatelet therapy in light of its clinical efficacy. A potential clinical benefit was assumed.

## 5. Endothelialization

Cellular coverage of synthetic and biologic surfaces is a valuable strategy to improve biocompatibility of implantable biomaterials [55]. This includes the usage of different cell types such as fibroblastic cells, macrophages, endothelial cells, or smooth muscle cells. However, variable results in animal studies and clinical trials require more research on tissue-engineered biomaterials [56]. Under physiological conditions, the endothelial cell provides a non-thrombogenic surface that does not allow platelets or other blood cells to adhere and does not activate the coagulation cascade [57] . This property of thromboresistance was used in regenerative medicine and cardiovascular tissue engineering for several decades. Endothelial cell seeding at the surface of synthetic and biological prosthesis is a valuable strategy to promote material biofunctionalization and to improve graft patency [55]. Two main seeding strategies were discussed: (1) *in vitro* and (2) self-endothelialisation of biomaterials. The main prerequisite for a successful endothelialization is a cell-adhesive biomaterial surface which allows adhesion/proliferation of viable autologous endothelial cells or progenitor cells [58]. 

### 5.1. In Vitro Endothelialization

*In vitro* endothelialization is based on the combination of biomaterials and culture expanded autologous vascular cells [59,60,61,62] – either endothelial cells alone or in coculture with fibroblasts and smooth muscle cells. Endothelial cell sources for tissue engineering are mature endothelial cells, endothelial progenitor cells or pluripotent stem cells [58]. Patient-derived cells could be isolated from vascular grafts such as a superficial vein, from a bone marrow punctuation, from peripheral blood and adipose tissue. Isolation of autologous vascular cells is limited due to several reasons, which include poor vessel quality, restricted proliferative capacity of harvested primary cells and technical difficulties to acquire pure populations of vascular cells. A prerequisite for complete coverage of the whole surface of artificial grafts with vascular cells is the expansion of the cells using bioreactors [63,64,65] or magnetic seeding [66]. In this context, several authors optimised shear stress conditions in the bioreactor to improve adhesive strength and cellular stability [67]. However, the composition of the underlying substrate is very influential in determining endothelial cell adhesion, proliferation and function. In this context, we demonstrated in a recent study that glutaraldehyde-crosslinked bovine pericardium could be endothelialized. However, there was no proliferative activity of the cells, but the anti-thrombotic and anti-inflammatory properties of the endothelial cell monolayer were conserved [68]. In addition, precoating with fibronectin and other extracellular matrix (ECM) proteins provides high levels of initial adhesion, influences cell shape, cytoskeletal organization, integrin binding, and controls proliferative and migratory behaviour of endothelial cells [58]. Fibronectin-coating is essential for the adhesion of endothelial cells while fibroblasts are able to produce their own ECM proteins to allow improved cell adhesion [69]. Coculture of fibroblasts and smooth muscle cells could also be used to produce ECM for stable endothelial cell seeding [67]. Another strategy to mask biophysical properties of polymeric materials was shown in our group [70]. Cell-repulsive materials such as polyethylene terephthalate, polypropylene, polytetrafluoroethylene (PTFE), polyurethane (PUR), and silicone were coated with a titaniumcarboxonitride layer using plasma-assisted chemical vapour deposition technique. This chemical modification overlaid the cell-repulsive properties of the polymers and allowed a complete endothelialization without affecting the function of the cells [70]. 

To date all procedures described for *in vitro* endothelialization are cost intensive, time-consuming and require special laboratory hardware and expertise for extraction and expansion for human applications. Therefore, in cardiovascular surgery the *in vitro* endothelialization is only practical before elective surgical interventions. *In vitro* endothelialization seems to be a relevant clinical application in heart valve and vascular graft replacement surgery. While *in vitro* endothelialization of artificial synthetic and biological grafts was successful in animal models such as sheep, calf or pig, in humans, prosthetic grafts remain largely without an endothelium. Repopularization of decellularized valves with autologous endothelial cells using a dynamic pulsatile bioreactor under simulated physiological conditions remained cellularized after three months of implantation in lambs. Thrombotic and neointima formations as well as calcification were observed in the decellularized valve types [71]. Due to the limited cell expansion capacity of these primary cells endothelial progenitor cell (EPC)-derived endothelial cells were isolated and expanded for seeding of heart valve matrices in an optimised bioreactor system [72]. This group demonstrated that preconditioning of EPCs seeded on valve matrices using a bioreactor system is necessary for achieving uniform endothelialization of valve scaffolds, which may reduce thrombotic activity after implantation *in vivo*. However, clinical application failed at the moment. The concept of *in vitro* endothelial seeding was also used in the field of arterial vascular reconstruction with small-diameter artificial grafts. Herring [73] and Graham [74] laid the foundation for endothelial seeding into the meshwork of synthetic vascular grafts. After 30 years of research in this area, precoating protocols including chemical coatings (collagen, fibronectin, laminin, poly-L-lysine, gelatin and ECM), pre-clotting (plasma, blood, serum and fibrin glue), chemical bonding (heparin, RGD and lectins) and other surface modifications as well as shear stress preconditioning and electrostatic charging improved the adhesion of the cells to vascular graft materials and increased graft patency. However, there is a discrepancy in the successful endothelialization of vascular grafts between animal and clinical studies. In search for an explanation for this discrepancy, insufficient seeding densities on the many times longer clinical grafts seemed plausible [75]. In addition, the experimental concept was queried [76]. A clinical update was done in 2005 by Bordenave *et al*. [4]. They reviewed controversial and mostly disappointing experimental and clinical trials. Neither “single-stage” seeding of freshly extracted endothelial cells onto expanded polytetrafluoroethylene (ePTFE) grafts, nor “two-stage” seeding of the same cells onto a fibrin-arginine-glycine-aspartate (RGD) tripeptide-enriched ePTFE graft in a rotating bioreactor could be used in vascular vessel replacement trials [77]. It was speculated that extracted endothelial cells were washed off the graft surface once exposed to blood flow. Another two-stage seeding strategy is the coculture of fibroblasts and endothelial cells onto ePTFE grafts. Usage of these constructs as arterial equivalent in a dog model demonstrated a doubling of the cell retention as well as a significant reduction in platelet adhesion and intimal hyperplasia response [78]. Furthermore, *in vitro* endothelialization of biodegradable tubular scaffolds fabricated from poly-L-lactic acid mesh coated with epsilon-caprolactone and L-lactide copolymer and implantation as infrarenal aortic interposition graft in a mutant mice strain with reduced natural killer cell activity (SCID-beige mice) resulted in an extensive remodelling with endothelial inner lining, and neomedia formation [79]. It was also shown in animal models, that bone marrow cells as a source for seeding onto a biodegradable scaffold are useful [80]. However, in any case, there was no clinical correlate. 

### 5.2. In Situ Endothelialization

In the case of emergency immediate implantation of biocompatible materials such as vascular grafts for peripheral vascular and coronary bypass surgery or implantation of ventricular assist devices is required. Hence, materials that promote in situ endothelialization would be highly desirable [58]. As shown above, endothelial seeding capacity depends on the surface characteristics of the underlying substrate. It has been known for more than forty years that in humans, transanastomotic endothelial ingrowth does not exceed more than 1–2 cm even after years of implantation [76] . Therefore, capture strategies are necessary to guarantee quick *in vivo* endothelialization after implantation of the prosthetic grafts. Relevant endothelial cell sources are circulating endothelial cells, endothelial progenitor cells [81], and progenitor cells, characterised by their cluster of differentiation as mesenchymal stem cells or endothelial progenitor cells [82,83,84]. The intention is to recruit the cells from the blood and immobilize the cells immediately onto the graft surface. The seeded cells should proliferate and differentiate to construct anti-thrombotic surfaces and functional tissues. Especially EPCs and bone marrow derive monocyte lineage cells have emerged as promising sources for prosthetic graft seeding [85]. De Mel *et al.* [55] summarized potential bioresponsive molecular components that can be incorporated into biomaterial surfaces to obtain accelerated, spontaneous, in situ endothelialization of vascular grafts. They enumerated many peptides/proteins and various factors and numerous techniques that could be used for biofunctionalization of biomaterials. 

In our present review we will first of all focus on the interactions between cell receptors and ECM. Protein adsorption and immediate cell attachment/behaviour is a desired process, which is determined by a variety of material properties such as surface chemistry, topography, dissolution rate, and the micro/macro mechanical elasticity. *In vivo* cells are in direct contact with the ECM. Therefore, some scientific approaches used ECM proteins or peptide sequences to improve cell attachment on the biomaterial. It was shown that collagene, fibronectin, laminin and vitronectin facilitate cell attachment. These proteins include intrinsic biological recognition sites for cells *via* integrin receptors [86]. Defined synthesized oligopeptides representing specific binding sites of the biofunctional domains of these proteins were also used in cardiovascular tissue engineering. Surfaces of polycarbonate, polyurethane urea and nanocomposite polymers were modified with RGD sequences to enhance cell adhesion [77,87]. In addition, a recombinant RGD-fusion protein increased cell adhesion as well as inhibited platelet activation on polyurethane materials [88]. Tang *et al.* [89] designed a surface modification for ePTFE consisting of a self-assembling fluorosurfactant polymer (FSP) bearing biologically active ligands. The choice of ligands presented in a FSP surface modification allows a selective adherence of endothelial cells with limited platelet attachment. An overview of the potential of these and other peptide sequences was presented in a review from De Mel *et al.* [55]. The geometric spatial arrangement of the ligands [90,91], ligand density [92,93], orientation and conformation [94] as well as stereochemistry of the sequence [95] determine the specificity for cell binding. In this context, it was shown that endothelial cells bind immobilized cyclic RGD more effectively than linear RGD [96]. Obviously, the cyclic peptide mimics the conformation of the native ligand which might be beneficial for *in situ* endothelialization. The cyclic RGD peptides could also act as potent, selective antagonists for the platelet integrin receptor (=GPIIb/IIIa) which is responsible for platelet aggregation (see section 3.). The specific inhibition of this receptor seems to avoid platelet-mediated thrombotic processes [97]. The specific role of integrins that govern the adhesive interactions between components of the ECM and cells *via* binding motifs was reviewed by de Mel *et al.* [55].

The latter described cyclic RGD peptides were coated on the polymer graft surfaces and work as capture molecules for circulating cells. Other capture strategies were published recently [98]. Avci-Adali *et al.* [98] discussed cyclic RGD peptides and three more approaches to fish out EPCs from bloodstream: Coating polymers with EPC antibodies, magnetic molecules and aptamers. In 2003 Kutryk *et al.* presented the first stent coating with murine monoclonal anti-human CD34 antibodies for capturing the EPCs from blood stream [99]. It was shown that after implantation of this device a functional endothelial cell layer is established in 1–2 days [99]. Promising results might allow a rapid discontinuity of the double antiplatelet therapy. However, the implantation of this EPC capture stent in a patient resulted in a late stent thrombosis and re-stenosis after withdrawal of clopidogrel [100]. Wendel *et al*. [101] elucidated the problems about this cell capturing stent. Only one of 250 CD34 positive cells is an EPC. In addition, not only endothelial cell derived EPCs were captured. A lot of other cells might be attracted which can differentiate into inflammatory cells or vascular smooth muscle cells. The efficacy of EPC capture stents might increase with the immobilization of a kinase insert domain receptor (KDR). The selectivity of the receptor was demonstrated *in vitro* in a model system under low shear rates [102]. However, neither animal nor clinical data were available. Another procedure to attract circulating EPCs at the site of a stented vessel wall is the use of magnetic force [103,104]. This technique requires the isolation and magnetic labelling of cells *in vitro*. The advantage of this method is the usage of a defined and homogenous cell phenotype which allows a controlled cell trafficking by magnetic resonance imaging. Aptamers are single-stranded nucleic acids that can bind with high affinity and specificity to a wide range of target molecules such as peptides, proteins, drugs, organic and inorganic molecules and even whole cells [101,105]. Hoffmann *et al.* [106] generated aptamers with a high affinity to circulating porcine EPCs. A porcine *in vitro* model was used to demonstrate the specific adhesion of EPCs and their differentiation into vital endothelial-like cells within 10 days in cell culture. This new technology may be useful to significantly increase the attachment of circulating EPCs from bloodstream to the blood contacting implants which might improve the clinical application of medical implants and tissue engineering strategies.

## 6. Conclusions

Thromboembolic complications of cardiovascular biomaterials make clinical applications of artificial materials often very difficult. This includes problems after usage of small-diameter cardiovascular vessel grafts, heart valve prosthesis, biocompatible circuit lines for hemodialysis and extracorporeal circulation as well as ventricular assist devices. Improved bio- and hemocompatibility of the prosthetic biomaterials is essential for successful treatment of cardiovascular diseases, the predominant diseases responsible for morbidity and mortality in the western world. In spite of the development of different strategies and encouraging *in vitro* results, to date no method attained clinical acceptance with seminal benefit for cardiac patients. One promising scientific approach seems to be the endothelialization of biomaterials. Therefore, future research may focus on the applicability of endothelialization as well as on the development and optimization of promising surface modification.

## References

[1] Vogler E.A., Siedllecki C.A. (2009). Contact activation of blood plasma coagulation. Biomaterials.

[2] Von Segesser L.K. (1996). Heparin-bonded surfaces in extracorporeal membrane oxygenation for cardiac support. Ann. Thorac. Surg..

[3] Hsu L.C. (2001). Heparin-coated cardiopulmonary bypass circuits: Current status. Perfusion.

[4] Bordenave L., Fernandez P., Remy-Zolghadri M., Villars S., Daculsi R., Midy D. (2005). *In vitro* endothelialized ePTFE prostheses: Clinical update 20 years after the first realization. Clin. Hemorheol. Microcirc..

[5] Schmaier A.H. (2008). The elusive physiologic role of factor XII. J. Clin. Invest..

[6] Colman R.W., Schmaier A.H. (1997). Contact system: A vascular biology modulator with anticoagulant, profibrinolytic, antiadhesive, and proinflammatory attributes. Blood.

[7] Colman R.W., Colman R.W., Marder V.J., Hirsh J., Clowes A.W. (2000). Contact activation pathway: Inflammatory fibrinolytic, anticoagulant, antiadhesive, and antiangiogenic activities. Hemostasis and Thrombosis: Basic Principles and Clinical Practice.

[8] Samuel M., Pixley R.A., Villanueva M.A., Colman R.W., Villanueva G.B. (1992). Human factor XII (Hageman factor) autoactivation by dextran sulfate. Circular dichroism, fluorescence, and ultraviolet difference spectroscopic studies. J. Biol. Chem..

[9] Hanson S.R. (1993). Device thrombosis and thromboembolism. Cardiovasc. Pathol..

[10] Ratner B.D. (1993). The blood compatibility catastrophe. J. Biomed. Mater. Res..

[11] Gorbet M.B., Sefton M.V. (2004). Biomaterial-associated thrombosis: roles of coagulation factors, complement, platelets and leukocytes. Biomaterials.

[12] Furie B., Furie B.C. (2008). Mechanism of thrombus formation. N. Eng. J. Med..

[13] Godo M.N., Sefton M.V. (1999). Characterization of transient platelet contacts on a polyvinyl alcohol hydrogel by video microscopy. Biomaterials.

[14] Sheppard J.I., McClung W.G., Feuerstein I.A. (1994). Adherent platelet morphology on adsorbed fibrinogen: Effects of protein incubation time and albumin addition. J. Biomed. Mater. Res..

[15] Park J.H., Bae Y.H. (2002). Hydrogels based on poly(ethylene oxide) and poly(tetramethylene oxide) or poly(dimethyl siloxane): Synthesis, characterization, *in vitro* protein adsorption and platelet adhesion. Biomaterials.

[16] Gemmell C.H., Ramirez S.M., Yeo E.L., Sefton M.V. (1995). Platelet activation in whole blood by artificial surfaces: Identification of platelet-derived microparticles and activated platelet binding to leukocytes as material-induced activation events. J. Lab. Clin. Med..

[17] Cholakis C.H., Zingg W., Sefton M.V. (1989). Effect of heparin-PVA hydrogel on platelets in a chronic canine AV shunt. J. Biomed. Mater. Res..

[18] Hanson S.R., Harker L.A., Ratner B.D., Hoffman A.S. (1980). *In vivo* evaluation of artificial surfaces with a nonhuman primate model of arterial thrombosis. J. Lab. Clin. Med..

[19] Ip W.F., Sefton M.V. (1991). Platelet consumption by NHLBI reference materials and silastic. J. Biomed. Mater. Res..

[20] Grunkemeier J.M., Tsai W.B., McFarland C.D., Horbett T.A. (2000). The effect of adsorbed fibrinogen, fibronectin, von Willebrand factor and vitronectin on the procoagulant state of adherent platelets. Biomaterials.

[21] Wu Y., Simonovsky F.I., Ratner B.D., Horbett T.A. (2005). The role of adsorbed fibrinogen in platelet adhesion to polyurethane surfaces: a comparison of surface hydrophobicity, protein adsorption, monoclonal antibody binding, and platelet adhesion. J. Biomed. Mater. Res. A.

[22] Wachtfogel Y.T., Hack C.E., Nuijens J.H., Kettner C., Reilly T.M., Knabb R.M., Bischoff R., Tschesche H., Wenzel H., Kucich U., Edmonds L.H., Colman R.W. (1995). Selective kallikrein inhibitors alter human neutrophil elastase release during extracorporeal circulation. Am. J. Physiol. Heart Circ. Physiol..

[23] Simon P., Ang K.S., Cam G. (1987). Enhanced platelet aggregation and membrane biocompatibility: Possible influence on thrombosis and embolism in haemodialysis patients. Nephron.

[24] Hakim R.M., Schafer A. (1985). Hemodialysis-associated platelet activation and thrombocytopenia. Am. J. Med..

[25] Jordan S.W., Chaikof E.L. (2007). Novel thromboresistant materials. J. Vasc. Surg..

[26] Merrill E.W., Salzman E.W. (1983). Polyethylene oxide as a biomaterial. ASAIO J..

[27] George P.A., Donose B.C., Cooper-White J.J. (2009). Self-assembling polystyrene-block-poly(ethylene oxide) copolymer surface coatings: resistance to protein and cell adhesion. Biomaterials.

[28] Debski R., Borovetz H., Haubold A., Hardesty R. (1982). Polytetrafluoroethylene grafts coated with Ulti carbon. Trans. Am. Soc. Artif. Intern. Organs.

[29] Prunotto M., Isaia C., Gatti M.A., Monari E., Pasquino E., Galloni M. (2005). Nitinol Carbofilm coated stents for peripheral applications: study in the porcine model. J. Mater. Sci. Mater. Med..

[30] Sick P.B., Gelbrich G., Kalnins U., Erglis A., Bonan R., Aengevaeren W., Elsner D., Lauer B., Woinke M., Brosteanu O., Schuler G. (2004). Comparison of early and late results of a carbofilm-coated stent versus a pure high-grade stainless steel stent (the carbostent-trial). Am. J. Cardiol..

[31] Bartorelli A.L., Trabattoni D., Montorsi P., Fabbiocchi F., Galli S., Ravagnani P., Grancini L., Cozzi S., Loaldi A. (2002). Aspirin alone antiplatelet regimen after intracoronary placement of the Carbostent (TM): The ANTARES study. Catheter. Cardiovasc. Interv..

[32] Sick P.B., Brosteanu O., Ulrich M., Thiele H., Niebauer J., Busch I., Schuler G. (2005). Prospective randomized comparison of early and late results of a carbonized stent versus a high-grade stainless steel stent of identical design: The Prevention of Recurrent Venous Thromboembolism (PREVENT) trial. Am. Heart J..

[33] Kim Y.H., Lee C.W., Hong M.K., Park S.W., Tahk S.J., Yang J.Y., Saito S., Santoso T., Quan L., Ge J., Weissman N.J., Lansky A.J., Mintz G.S., Park S.J. (2005). Randomized comparison of carbon ion-implanted stent versus bare metal stent in coronary artery disease: The Asian Pacific Multicenter Arthos Stent Study (PASS) trial. Am. Heart J..

[34] Kornowski R. (2009). A critical appraisal of the Janus carbostent. Catheter Cardiovasc. Interv..

[35] Arabi H., Mirzadeh H., Ahmadi S.H., Amanpour S., Rabbani S., Abdi A. (2004). *In vitro* and *in vivo* hemocompatibility evaluation of graphite coated polyester vascular grafts. Int. J. Artif. Organs.

[36] Tegoulia V.A., Rao W.S., Kalambur A.T., Rabolt J.R., Cooper S.L. (2001). Surface properties, fibrinogen adsorption, and cellular interactions of a novel phosphorylcholine-containing self-assembled monolayer on gold. Langmuir.

[37] Lu J.R., Murphy E.F., Su T.J., Lewis A.L., Stratford P.W., Satija S.K. (2001). Reduced protein adsorption on the surface of a chemically grafted phospholipid monolayer. Langmuir.

[38] Glasmastar K., Larsson C., Hook F., Kasemo B. (2002). Protein adsorption on supported phospholipid bilayers. J. Colloid. Interface Sci..

[39] Andersson A.S., Glasmastar K., Sutherland D., Lidberg U., Kasemo B. (2003). Cell adhesion on supported lipid bilayers. J. Biomed. Mater. Res. A.

[40] Jordan S.W., Faucher K.M., Caves J.M., Apkarian R.P., Rele S.S., Sun X.L., Hanson S.R., Chaikof E.L. (2006). Fabrication of a phospholipid membrane-mimetic film on the luminal surface of an ePTFE vascular graft. Biomaterials.

[41] Barnes M.J., Macintyre D.E. (1979). Platelet-reactivity of isolated constituents of the blood-vessel wall. Haemostasis.

[42] Jordan S.W., Haller C.A., Sallach R.E., Apkarian R.P., Hanson S.R., Chaikof E.L. (2007). The effect of a recombinant elastin-mimetic coating of an ePTFE prosthesis on acute thrombogenicity in a baboon arteriovenous shunt. Biomaterials.

[43] Defife K.M., Hagen K.M., Clapper D.L., Anderson J.M. (1999). Photochemically immobilized polymer coatings: Effects on protein adsorption, cell adhesion, and leukocyte activation. J. Biomater. Sci. Polym. Ed..

[44] Sperling C., Schweiss R.B., Streller U., Werner C. (2005). *In vitro* hemocompatibility of self-assembled monolayers displaying various functional groups. Biomaterials.

[45] Laredo J., Xue L., Husak V.A., Ellinger J., Greisler H.P. (2003). Silyl-heparin adsorption improves the *in vivo* thromboresistance of carbon-coated polytetrafluoroethylene vascular grafts. Am. J. Surg..

[46] Luong-Van E., Grondahl L., Chua K.N., Leong K.W., Nurcombe V., Cool S.M. (2006). Controlled release of heparin from poly(epsilon-caprolactone) electrospun fibers. Biomaterials.

[47] Larm O., Larsson R., Olsson P. (1983). A new non-thrombogenic surface prepared by selective covalent binding of heparin *via* a modified reducing terminal residue. Biomater. Med. Devices Artif. Organs.

[48] Pasche B., Elgue G., Olsson P., Riesenfeld J., Rasmuson A. (1991). Binding of antithrombin to immobilized heparin under varying flow conditions. Artif. Organs.

[49] Elgue G., Blomback M., Olsson P., Riesenfeld J. (1993). On the mechanism of coagulation inhibition on surfaces with end-point immobilized heparin. Thromb. Haemost..

[50] Edmunds L.H., Colman R.W. (2006). Thrombin during cardiopulmonary bypass. Ann. Thorac. Surg..

[51] Kishida A., Ueno Y., Maruyama I., Akashi M. (1994). Immobilization of human thrombomodulin on biomaterials: Evaluation of the activity of immobilized human thrombomodulin. Biomaterials.

[52] Vasilets V.N., Hermel G., König U., Werner C., Müller M., Simon F., Grundke K., Ikada Y., Jacobasch H.J. (1997). Microwave CO2 plasma-initiated vapour phase graft polymerization of acrylic acid onto polytetrafluoroethylene for immobilization of human thrombomodulin. Biomaterials.

[53] Yeh H.Y., Lin J.C. (2009). Bioactivity and platelet adhesion study of a human thrombomodulin-immobilized nitinol surface. J. Biomater. Sci. Polym. Ed..

[54] Kidane A.G., Salacinski H., Tiwari A., Bruckdorfer K.R., Seifalian A.M. (2004). Anticoagulant and antiplatelet agents: their clinical and device application(s) together with usages to engineer surfaces. Biomacromolecules.

[55] De Mel A., Jell G., Stevens M.M., Seifalian A.M. (2008). Biofunctionalization of biomaterials for accelerated in situ endothelialization: a review. Biomacromolecules.

[56] Knight R.L., Wilcox H.E., Korossis S.A, Fisher J., Ingham E. (2008). The use of acellular matrices for the tissue engineering of cardiac valves. Proc. Inst. Mech. Eng. H.

[57] Bunting S., Moncada S., Vane J.R. (1977). Antithrombotic properties of vascular endothelium. Lancet.

[58] McGuigan A., Sefton M. (2007). The influence of biomaterials on endothelial cell thrombogenicity. Biomaterials.

[59] Seifalian A.M., Tiwari A., Rashid S.T., Salacinski H., Hamilton G. (2002). Impregnation of the the polymeric graft with adhesives molecules, typically oligopeptides or glycoprotein improves retention. Artif. Organs.

[60] Meinhart J.G., Deutsch M., Fischlein T., Howanietz N., Froschl A., Zilla P. (2001). Clinical autologous *in vitro* endothelialization of 153 infrainguinal ePTFE grafts. Ann. Thorac. Surg..

[61] Thomas A.C., Campbell G.R., Campbell J.H. (2003). Advances in vascular tissue engineering. Cardiovasc. Pathol..

[62] Thebaud N.B., Pierron D., Bareille R., Le Visage C., Letourneur D., Bordenave L. (2007). Human endothelial progenitor cell attachment to polysaccharide-based hydrogels: a pre-requisite for vascular tissue engineering. J. Mater. Sci. Mater. Med..

[63] Alobaid N., Salacinski H.J., Sales K.M., Hamilton G., Seifalian A.M. (2005). Single stage cell seeding of small diameter prosthetic cardiovascular grafts. Clin. Hemorheol. Microcirc..

[64] Wu Y.F., Zhang J., Gu Y.Q., Li J.X., Wang L.C., Wang Z.G. (2008). Reendothelialization of tubular scaffolds by sedimentary and rotative forces: a first step toward tissue-engineered venous graft. Cardiovasc. Revasc. Med..

[65] Teebken O.E., Puschmann C., Breitenbach I., Rohde B., Burgwitz K., Haverich A. (2009). Preclinical development of tissue-engineered vein valves and venous substitutes using re-endothelialised human vein matrix. Eur. J. Vasc. Endovasc. Surg..

[66] Perea H., Aigner J., Hopfner U., Wintermantel E. (2006). Direct magnetic tubular cell seeding: a novel approach for vascular tissue engineering. Cells Tissues Organs.

[67] Gulbins H., Pritisinac A., Petzold R., Goldemund A., Doser M., Dauner M., Meiser B., Reichart B., Daebritz S. (2005). A low-flow adaptation phase improves shear-stress resistance of artificially seeded endothelial cells. Thorac. Cardiovasc. Surg..

[68] Schopka S., Schmid F.X., Hirt S., Birnbaum D.E., Schmid C., Lehle K. (2009). Recellularization of biological heart valves with human vascular cells: *in vitro* hemocompatibility assessment. J. Biomed. Mater. Res. B Appl. Biomater..

[69] Lehle K., Stock M., Schmid T., Schopka S., Straub R.H., Schmid C. (2009). Cell-type specific evaluation of biocompatibility of commercially available polyurethanes. J. Biomed. Mater. Res. B Appl. Biomater..

[70] Lehle K., Buttstaedt J., Birnbaum D.E. (2003). Expression of adhesion molecules and cytokines *in vitro* by endothelial cells seeded on various polymer surfaces coated with titaniumcarboxonitride. J. Biomed. Mater. Res. A.

[71] Lichtenberg A., Tudorache I., Cebotari S., Suprunov M., Tudorache G., Goerler H., Park J.K., Hilfiker-Kleiner D., Ringes-Lichtenberg S., Karck M., Brandes G., Hilfiker A., Haverich A. (2006). Preclinical testing of tissue-engineered heart valves re-endothelialized under stimulated physiological conditions. Circulation.

[72] Lee D.J., Steen J., Jordan J.E., Kincaid E.H., Kon N.D., Atala A., Berry J., Yoo J.J. (2009). Endothelialization of heart valve matrix using a computer.assisted pulsatile bioreactor. Tissue Eng. A.

[73] Herring M., Dilley R., Jersild R.J., Boxer L., Gardner A., Glover J. (1979). Seeding arterial prostheses with vascular endothelium. The nature of lining. Ann. Surg..

[74] Graham L., Burkel W., Ford J., Vinter D., Kahn R., Stanley J. (1980). Immediate seeding of enzymatically derived endothelium in Dacron vascular grafts. Early experimental studies with autologous canine cells. Arch Surg.

[75] Deutsch M., Meinhart J., Zilla P., Howanietz N., Gorlitzer M., Froeschl A., Stuempflen A., Bezuidenhout D., Grabenwoeger M. (2009). Long-term experience in autologous *in vitro* endothelialization of infrainguinal ePTFE grafts. J. Vasc. Surg..

[76] Zilla P., Bezuidenhout D., Human P. (2007). Prosthetic vascular grafts: Wrong models, wrong questions and no healing. Biomaterials.

[77] Alobaid N., Salacinski H.J., Sales K.M., Hamilton G., Seifalian A.M. (2005). Single stage cell seeding of small diameter prosthetic cardiovascular grafts. Clin. Hemorheol. Microcirc..

[78] Garcia-Honduvilla N., Dominguez B., Pascual G., Escudero C., Minguela F., Bellon J.M., Bujan J. (2008). Viability of engineered vessels as arterial substitutes. Ann. Vasc. Surg..

[79] Mirenski T.L., Nelson G.N., Brennan M.P., Roh J.D., Hibino N., Yi T., Shinoka T., Breuer C.K. (2009). Tissue-engineered arterial grafts: long.term results after implantation in a small animal model. J. Pediatr. Surg..

[80] Shinoka T., Breuer C. (2008). Tissue-engineered blood vessels in pediatric cardiac surgery. Yale J. Biol. Med..

[81] Melero-Martin J.M., Khan Z.A., Picard A., Wu X., Paruchuri S., Bischoff J. (2007). *In vivo* vasculogenic potential of human blood-derived endothelial progenitor cells. Blood.

[82] Urbich C., Dimmeler S. (2004). Endothelial progenitor cells: Characterization and role in vascular biology. Circ. Res..

[83] Jevon M., Dorling A., Hornick P.I. (2008). Progenitor cells and vascular disease. Cell Prolif..

[84] Krenning G., Moonen J.R., van Luyn M.J., Harmsen M.C. (2008). Generating new blood flow: integrating developmental biology and tissue engineering. Trends Cardiovasc. Med..

[85] Rothmans J.I, Heyligers J.M.M, Stroes E.S.G, Pasterkamo G. (2006). Endothelial progenitor cell-seeded grafts: Rash and risky. Can. J. Cardiol..

[86] Rosso F., Giordano A., Barbarisi M., Barbarisi A. (2004). From cell-ECM interactions to tissue engineering. J. Cell Physiol..

[87] Alobaid N., Salacinski H.J., Sales K.M., Ramesh B., Kannan R.Y., Hamilton G., Seifalian A.M. (2006). Nanocomposite containing bioactive peptides promote endothelialization by circulating progenitor cells: an *in vitro* evaluation. Eur. J. Vasc. EndoVasc. Surg..

[88] Hsu S.H., Chu W.P., Lin Y.S., Chiang Y.L., Chen D.C., Tsai C.L. (2004). The effect of an RGD-containing fusion protein CBD-RGD in promoting cellular adhesion. J. Biotechnol..

[89] Tang C., Kligman F., Larsen C.C., Kottke-Marchant K., Marchant R.E. (2009). Platelet and endothelial adhesion on fluorosurfactant polymers designed for vascular graft modification. J. Biomed. Mater. Res. A.

[90] Massia S.P., Hubbell J.A. (1991). An RGD spacing of 440 nm is sufficient for integrin alpha V beta 3-mediated fibroblast spreading and 140 nm for focal contact and stress fiber formation. J. Cell Biol..

[91] Koo L.Y., Irvine D.J., Mayes A.M., Lauffenburger D.A., Griffith L.G.J. (2002). Fibronectin matrix assembly regulates alpha5beta1-mediated cell cohesion. Cell Sci..

[92] Patel S., Tsang J., Harbers G.M., Healy K.E., Li S. (2007). Regulation of endothelial cell function by GRGDSP peptide grafted on interpenetrating polymers. J. Biomed. Mater. Res. A.

[93] Schense J.C., Hubbell J.A. (2000). Three-dimensional migration of neurites is mediated by adhesion site density and affinity. J. Biol. Chem..

[94] Koivunen E., Wang B.C., Ruoslahti E. (1995). Phage libraries displaying cyclic peptides with different ring sizes: ligand specificities of the RGD-directed integrins. Bio-Technology.

[95] Pierschbacher M.D., Ruoslahti E. (1987). Influence of stereochemistry of the sequence Arg-Gly-Asp-Xaa on binding specificity in cell adhesion. J. Biol. Chem..

[96] Xiao Y., Truskey G.A. (1996). Effect of receptor-ligand affinity on the strength of endothelial cell adhesion. Biophys. J..

[97] Cheng S., Craig W.S., Mullen D., Tschopp J.F., Dixon D., Pierschbacher M.D. (1994). Design and synthesis of novel cyclic RGD-containing peptides as highly potent and selective integrin alpha IIb beta 3 antagonists. J. Med. Chem..

[98] Avci-Adali M., Paul A., Ziemer G., Wendel H.P. (2008). New strategies for *in vivo* tissue engineering by mimicry of homing factors for self-endothelialization of blood contacting materials. Biomaterials.

[99] Kutryk M.J., Kuliszewski M.A. (2003). *In vivo* endothelial progenitor cell seeding for the accelerated endothelialisation of endovascular devices. Am. J. Cardiol..

[100] Rossi M.L., Zavalloni D., Gasparini G.L., Mango R., Belli G., Presbitero P. (2009). The first report of late stent thrombosis leading to acute myocardial infarction in patient receiving the new endothelial progenitor cell capture stent. Int. J. Cardiol..

[101] Wendel H.P., Avci-Adali M., Ziemer G. (2009). Endothelial progenitor cell capture stents - hype or hope?. Int. J. Cardiol..

[102] Markway B.D., McCarty O.J., Marzec U.M., Courtman D.W., Hanson S.R., Hinds M.T. (2008). Capture of flowing endothelial cells using surface-immobilized anti-kinase insert domain receptor antibody. Tissue Eng. C Methods.

[103] Consigny P.M., Silverberg D.A., Vitali N.J. (1999). Use of endothelial cells containing superparamagnetic microspheres to improve endothelial cell delivery to arterial surfaces after angioplasty. J. Vasc. Interv. Radiol..

[104] Pislaru S.V., Harbuzariu A., Agarwal G., Witt T., Gulati R., Sandhu N.P., Mueske C., Kalra M., Simari R.D., Sandhu G.S. (2006). Magnetic forces enable rapid endothelialization of synthetic vascular grafts. Circulation.

[105] Guo K.T., Schafer R., Paul A., Gerber A., Ziemer G., Wendel H.P. (2006). A new technique for the isolation and surface immobilization of mesenchymal stem cells from whole bone marrow using high-specific DNA aptamers. Stem Cells.

[106] Hoffmann J., Paul A., Harwardt M., Groll J., Reeswinkel T., Klee D., Moeller M., Fischer H., Walker T., Greiner T., Ziemer G., Wendel H.P. (2008). Immobilized DNA aptamers used as potent attractors for porcine endothelial precursor cells. J. Biomed. Mater. Res. A.

